# Multi-route human health risk assessment from trihalomethanes in drinking and non-drinking water in Abadan, Iran 

**DOI:** 10.1007/s11356-020-09990-9

**Published:** 2020-07-25

**Authors:** Raheleh Kujlu, Mostafa Mahdavianpour, Farshid Ghanbari

**Affiliations:** Department of Environmental Health Engineering, Abadan Faculty of Medical Sciences, Abadan, Iran

**Keywords:** Abadan, Drinking water, Disinfection by-products, Risk assessment, Trihalomethanes

## Abstract

Natural organic matter reacted with chlorine used for disinfection, and finally, trihalomethanes (THMs) are formatted. The main purpose of this study was to determine four THM concentrations and human health cancer risk and non-cancer risk assessment from exposure through oral ingestion, dermal contact, and inhalation for males and females in Abadan. Two sampling sites were selected, and five samples before and after treatment by two different water treatment systems (RO and ion exchange) were collected every week. Results showed that total THM concentrations before and after treatment by RO were 98.1 and 8.88 μg/L, and ion exchange ranged between 101.9 and 14.96 μg/L, respectively, that before treatment was upper than the maximum of 80 mg/L recommended by USEPA. Inhalation was the primary route of exposure by around 80–90% of cancer risk. Total cancer risk was higher than the USEPA acceptable limit of 10^−6^ via three exposure routes. Oral route has the higher hazard index values than dermal ways.

## Introduction

Providing safe water is a significant concern in public health. To achieve this important goal and to prevent secondary pollution, disinfectants are used during the last step of water treatment. Chlorine, chloramines, ozone, and ClO_2_ are general disinfectants. They can generate disinfection by-products (DBPs) that can take a risk to human health (Al-Otoum et al. 2016). Among all disinfection methods, chlorination is used frequently because of low costs, effectiveness, and simple operation (Arman et al. 2016; Wang et al. 2007). Chlorine secure human health risk from waterborne diseases, deactivated microorganism and immune bacteria, virus, and protozoa regrowth in the water distribution system (Pentamwa et al. 2013). Chlorine interacts with organic matter especially humic and fulvic acids, and up to 700 species of DBPs are produced (Genisoglu et al. 2019; Grellier et al. 2015; Pan et al. 2014). Among them, trihalomethanes (THMs) are the most critical disinfectant by-products (Genisoglu et al. 2019; Golea et al. 2017; Mohammadi et al. 2016; Wang et al. 2019). THMs are include chloroform (CHCl_3_), dichlorobromomethane (CHCl_2_Br), chlorodibromomethane (CHClBr_2_), and bromoform (CHBr_3_) (Arman et al. 2016). According to the United States Environmental Protection Agency (USEPA), these compounds are categorized as groups B1, B2, and C (Wang et al. 2019). Several researches showed that DBPs can enhance the hazard of some gastrointestinal cancers, reproductive disorders, miscarriages, low birth weights and pre-term deliveries, and neural tube defects (Kumari et al. 2015; Siddique et al. 2015; Wang et al. 2019; Yang et al. 2019).

**Fig. 1:**
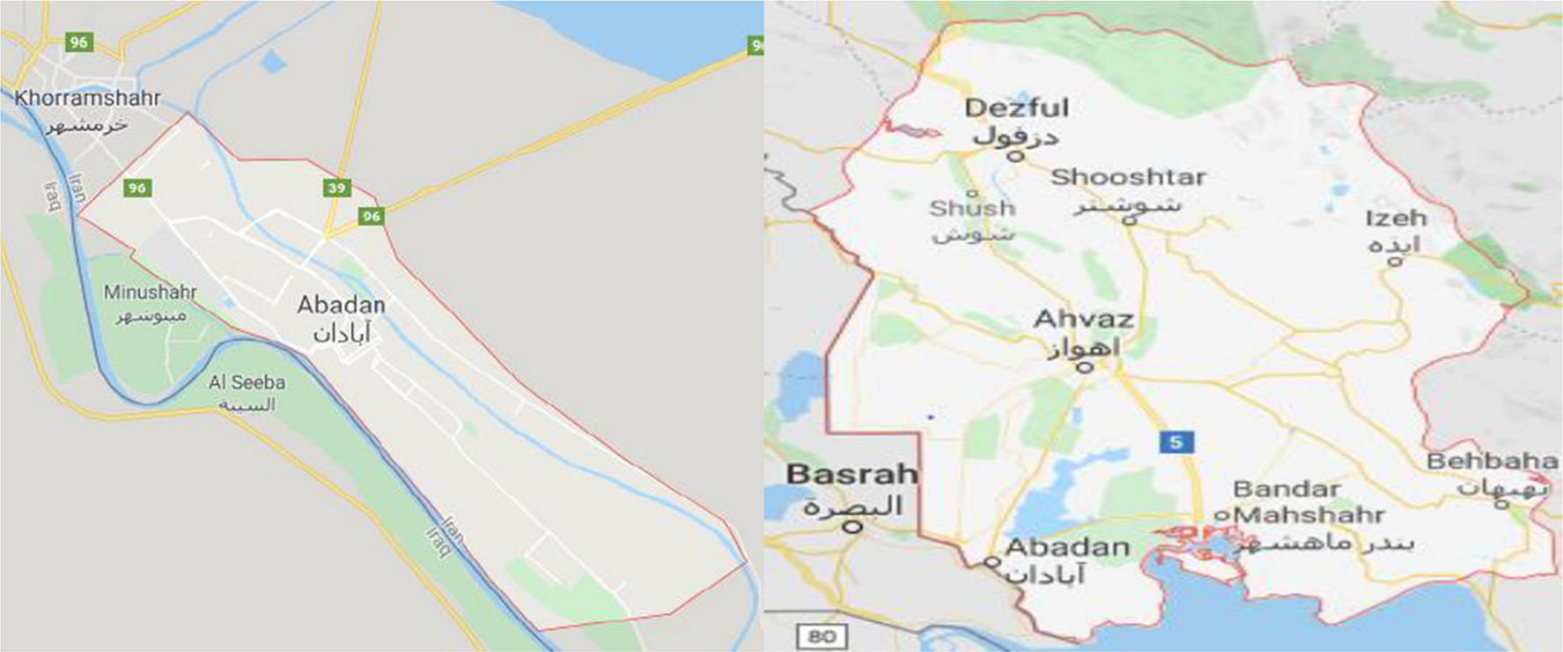
The location of sampling sites in the present study

Water quality can affect DBPs production (because high organic matters in surface water generate more THMs compared to groundwater) (Amjad et al. 2013). According to the United States Environmental Protection Agency (USEPA) and Europe Community (EC), the highest acceptable amount of THMs in drinking water is 80 and 100 μg/L, respectively (Wang et al. 2019). Chlorine-treated water is used for drinking and regular daily activated hand washing, showering, swimming, cleaning, or cooking. Potential exposure pathway occurs by ingestion, dermal contact, and inhalation (Hrudey 2009). Although many studies mentioned that ingestion is the primary way for exposure to THMs, there is also a potential for exposure through dermal absorption and inhalation. Studies show that exposure to volatile THMs from dermal and inhalation route may be larger than oral consumption. Abadan is located in Khuzestan, south-west of Iran (Fig. 1) that suffers from freshwater resources. Arvand and Bahmanshir Rivers are two important water sources in Abadan. Due to the presence of industries and other pollutants near the rivers and discharge of domestic wastewater, the potential of THMs formation is apparent. Several studies estimated the health risk assessments of THMs through multi-pathway exposure routes in chlorinated drinking water. For example, it was reported that chloroform was the most common THMs in chlorinated water in Pakistan and the main way of exposure was through oral ingestion (Amjad et al. 2013). Wang et al. calculated cancer risk assessment from trihalomethanes in drinking water; results revealed that the highest risk comes from the inhalation exposure to chloroform during showers (Wang et al. 2007). Cancer risk assessment was performed in drinking water from ten water treatment plants in China; the results showed that the oral ingestion was the main route of exposure (Gan et al. 2013). A similar study was conducted for cancer risk assessment from exposure to chloroform in drinking water in Ilam, Iran, and it was reported that the oral ingestion was the main route of exposure, followed by inhalation and dermal absorption (Arman et al. 2016). Also, the higher cancer risk was reported through inhalation followed by dermal route in Ahvaz, Iran (Babaei et al. 2015). NO study has been managed to follow THMs in the drinking water supplies in Abadan, Iran.

In this city, water containing high total dissolved solids (TDS) is pumped into the distribution network after conventional water treatment, so household water purifiers are used in the point of use. Most of these devices contained reverse osmosis (RO) membrane and ion exchange unit in their modules. The main aim of this study was to measure THMs concentration before and after point-of-use water treatment devices. The second purpose was to assess THMs health risks for both males and females from multi-exposure route; oral exposure for treated water by point of use water treatment devices and inhalation and dermal exposure for untreated water.

## Materials and methods

### Study area and water characteristics

Abadan is one of the most important cities in Khuzestan province (Iran), with more than 300,000 population located at the Southwest in Iran (Fig. 1). This city has 2538-km^2^ areas. The major resource of drinking water supply in Abadan is surface water from Bahmanshir and Arvand Rivers. There are a lot of points and nonpoint sources of pollution which enter to these rivers. The water treatment plant system has conventional treatment units including sedimentation, coagulation-flocculation, filtration, and disinfection with chlorine. Two sampling sites were selected in the Abadan faculty of medical sciences (school and Ghonooti dormitory). Two point-of-use water treatment devices were used in two sampling locations (school and dormitory) containing RO membrane and ion exchange in their chains, respectively.

### Data sampling and analysis

Water samples were collected before and after two different water treatment systems (RO and ion exchange, here) for 5 weeks. Samples were collected in 100-ml glass vial from each location according to EPA method (EPA 2005). Ascorbic acid (0.2 g) was added to vials to stop THMs formation and eliminate remaining residual chlorine. Samples were stored in a cold place (4 ^o^C) before analysis (Wang et al. 2019). Temperature, pH and conductivity were measured by pH meter and EC meter (8603 Mettler Toledo).

### Determination of THMs and TOC

Liquid-liquid extraction was conducted by mixing 4 mL of MTBE (extraction solvent) with 20 mL of the water sample. Six grams of anhydrous sodium sulfate was added to the mixture and shaken for 5 min. One milliliter of the upper organic layer was transferred to a vial to analyze THMs. The THMs concentration was determined by gas chromatography (GC) (Agilent) equipped with an ECD (electron capture detector). The THMs mixure solution was purchased with a concentration of 1000 mg/L for calibration. The DB-5 capillary column (30.0 m × 0.32 mm × 0.25 μm nominal) was used for peak separation. The injection volume to the column was 2 μL. The carrier gas was helium at a rate of 1 mL/min. The injector and detector temperature were 230 and 250 °C, respectively. Total organic carbon (TOC) was determined by TOC analyzer (Shimadzu). Residual chlorine was determined by the diethyl paraphenylene diamine (DPD) method.

### Cancer risk assessment

According to USEPA guideline, cancer risk assessment for THMs in drinking and non-drinking water was estimated through oral ingestion, dermal contact, and inhalation absorption (Assessment 1992). Based on THMs concentration, cancer risk was estimated using chronic daily intake (CDI) and the slope factor (SF). CDI value for different routes of exposure was computed using equations 1–3 (Amjad et al. 2013; Pardakhti et al. 2011; Siddique et al. 2015; Wang et al. 2007). Cancer risk is defined in four classes: negligible risk (CR < 10^−6^), acceptable low risk (1 × 10^−6^ ≤ CR < 5.1 × 10^−5^), acceptable high risk (5.1 × 10^−5^ ≤ CR < 10^−4^), and unacceptable risk (CR ≥ 10^−4^) (Legay et al. 2011).

1 $$ {\mathrm{CDI}}_{\mathrm{inge},\mathrm{i}}=\frac{C_{\mathrm{wi}}\times \mathrm{IR}\times \mathrm{EF}\times \mathrm{ED}\times \mathrm{CF}}{\mathrm{BW}\times \mathrm{AT}} $$

2 $$ {\mathrm{CDI}}_{\mathrm{derm},\mathrm{i}}=\frac{C_{\mathrm{wi}}\times \mathrm{SA}\times \mathrm{Kpi}\times \mathrm{ET}\times \mathrm{EF}\times \mathrm{ED}}{\mathrm{BW}\times \mathrm{AT}} $$

3 $$ {\mathrm{CDI}}_{\mathrm{inha},\mathrm{i}}=\frac{C_{\mathrm{ai}}\times \mathrm{IRa}\times \mathrm{ET}\times \mathrm{EF}\times \mathrm{ED}}{\mathrm{BW}\times \mathrm{AT}} $$

where CDI is the chronic daily intake through oral, dermal and inhalation exposure (mg/kg/day), *C*_wi_ is the DBPs concentration in drinking water (mg/L), IR is the ingestion rate of treated water (L/day), EF is the exposure frequency (day/year), ED is the exposure duration (years), BW is the body weight (kg), AT is the average exposure time (days), SA is the skin surface area exposed to THMs (cm^2^), Kp is the specific dermal permeability constant (cm/h), ET is the exposure time (h/event), CF is the conversion factor of 10^−3^ (L/cm^3^), Ca is the concentration of THMs in air (mg/m^3^), and IRa is the inhalation rate (m^3^/h).

Some of these parameters values are shown in Table 1. For inhalation intake, a model developed by Little (1992) (equations (4–8) was used for estimating THM concentrations in air (Ca). Specific contents for estimation of THMs concentration in the air are shown in Table 2.

4 $$ Ys(t)=\left[1-\exp \left(\mathrm{bt}\right)\right]\left(\frac{a}{b}\right) $$

5 $$ b=\left\{\left(\frac{\mathrm{QL}}{H}\right)\left[1-\exp (N)\right]+\mathrm{QG}\right\}/ Vs $$

6 $$ a=\left\{\mathrm{QLCW}\left[1-\exp (N)\right]\right\}/ Vs $$

7 $$ N=\left(\mathrm{KOLA}\right)/ QL $$

8 $$ C\mathrm{a} ir=\left( Ys(t)+ Ysi\right)/2 $$

### Non-cancer risk assessment

Equations (9) and (10) are used to calculate hazard index (HI) for the determination of non-cancer risks of THMs via oral ingestion and dermal contact.

9 $$ \mathrm{oral}\ \mathrm{hazard}\ \mathrm{index}=\mathrm{CDI}\ \mathrm{oral}/\mathrm{RfD} $$

10 $$ \mathrm{dermal}\ \mathrm{hazard}\ \mathrm{index}=\mathrm{CDI}\ \mathrm{dermal}/\mathrm{RfD} $$

The values of reference dose (RfD)are shown in Table 1. Due to the fact that chronic daily intakes (CDI) from inhalation are very lower than oral ingestion and dermal exposure routes, inhalation absorption was neglected (Wang et al. 2019).

### THM concentrations in chlorinated water in Abadan

Water samples were collected from school and a dormitory before and after treatment by RO and ion exchange systems and analyzed for THMs concentration. The average values of obtained data are shown in Table 3 and compared with the World Health Organization (WHO) guideline values (200, 60, 100, and 100 mg/L) for CHCl_3_, CHCl_2_Br, CHClBr_2_, and CHBr_3_, respectively). Mean values for total THMs (TTHMs) concentration were 98.1 and 8.88 μg/L before and after treatment by RO in school, respectively, and their values were 101.8 and 14.96 μg/L before and after treatment with ion exchange in dormitory, respectively. The removal efficiency of trihalomethanes by RO was higher than that of ion exchange (90.9 and 85.3 μg/L, respectively), which was higher than the prescribed USEPA standards of 80 μg/L before treatment with RO and ion exchange in both sites. That is similar to other studies (Siddique et al. 2015). Generally, chloroform is the most dominant DBP in chlorinated drinking waters (Basu et al. 2011; Siddique et al. 2015) but in this study DBCM was identified as the highest concentration THM species befor both treatment systems, as shown in Table 3, and then, the bromoform has the highest concentration. This was because of the high concentration of bromide in water resources (Table 4).

### Effect of water quality parameters on THMs formation

The water quality parameters are presented in Table 4. The pH values for all water samples was in the normal range. TOC concentrations were also very low compared to data reported in other studies (Ahmed et al. 2019; Hassani et al. 2010; Zimoch and Stolarczyk 2010), with the average concentrations of 1.3 and 0.82 mg/L before water treatment by RO and ion exchange, respectively, and 0.142 and 0.108 mg/L after treatment by RO and ion exchange, indicating that RO was more efficient for removing TOC (89%). Free chlorine residual concentrations were 0.3 and 0.76 mg/L before treatment by RO and ion exchange, respectively, that was in the normal range for microbial inactivation. Bromide concentration was the same as 0.38 mg/L and removed to 0.03 and 0.058 mg/L after water treatment by RO and ion exchange, respectively. As before mentioned, bromide can affect the rate of THMs formation during chlorination, so that bromide is oxidized to bromine that reacts with organic matter and forms brominated THMs.

### Cancer risk analysis of THMs through different routes

#### Oral cancer risk

**Fig. 2:**
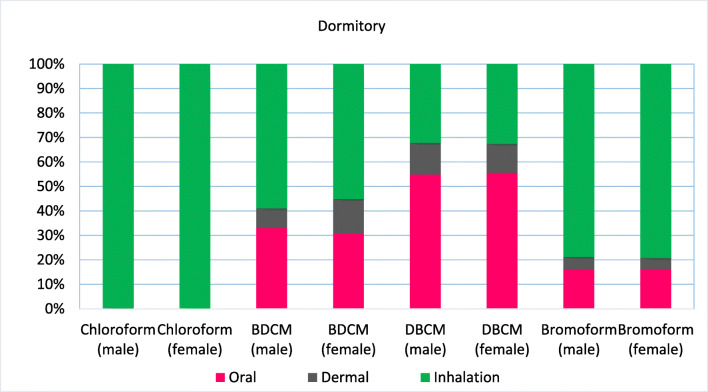
Cancer risk assessment of THMs via oral (after treatment with ion exchange), dermal, and inhalation (before treatment with ion exchange) for both sexes in the dormitory

**Fig. 3:**
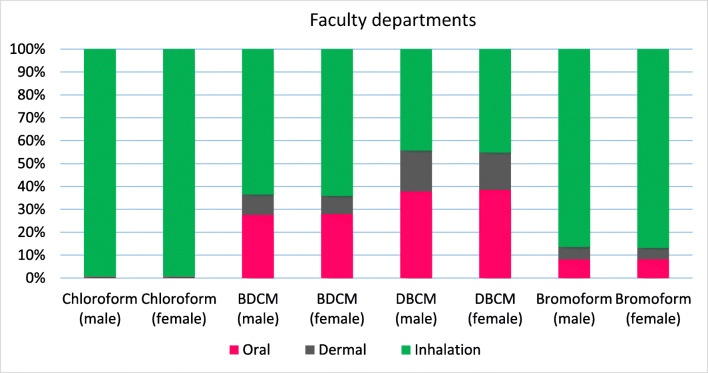
Cancer risk assessment of THMs via oral (after treatment with ion exchange), dermal, and inhalation (before treatment by ion exchange) for males and females in school

The carcinogenic risk for each type of THM compounds is shown in Figs.1, 2 and 3. Average lifetime cancer risk for TTHMs via ingestion in treated water samples was in the range of acceptable low risk (1 × 10^−6^ ≤ CR < 5.1 × 10^−5^), and the risk contribution was observed in the following arrangement: DBCM > BDCM > bromoform > chloroform. It was the same for males and females. Among four THMs, DBCM showed the highest oral cancer risk of 1.42529E-05 for males and 1.46768E-05 for females in the dormitory samples and 7.81609E-06 and 8.04858E-06 for males and females in school, respectively. This result confirms WHO report about cancer risks related to THMs (Abbas et al. 2015). The lowest oral cancer risk in both males and females was related to chloroform that was lower than 10^−6^ (negligible risk), which may be due to the increase existence of bromine compounds over-chlorinated compounds in water samples. Cancer risk for all THMs in the female was higher than males. In contrast with other studies, chloroform has the lowest cancer risk due to the low concentration in the water samples (Abbas et al. 2015; Lee et al. 2004). The highest contribution to the average lifetime cancer risk was related to DBCM (65%), followed by BDCM, bromoform, and chloroform with 30% and 3% and 2% in dormitory samples and DBCM (64%), BDCM (31%), bromoform (4%), and chloroform (1%) in school station.

**Table 1:** Parameter definition and their values which were used in this study

Type of parameter	Parameter	Symbol	Unit	Value	References
General	THMs concentration	*C*_wi_	μg/L	Table 3	This study
	Average lifetime	AT	Days	Male 64 years and female 66 years	Siddique et al. (2015)
	Body weight	BW	kg	65 kg (67.2 kg, female 63.9 kg)	Siddique et al. (2015)
	Exposure duration	ED	Year	Male 64 years and female 66 years	Siddique et al. (2015)
	Exposure frequency	EF	Days/year	365	Abbas et al. (2015)
Oral ingestion	Ingestion rate	IR	L/day	2	USEPA (1997)
Dermal	Area of surface skin expose to water	SA	m^2^	Male: 1.7 Female: 1.53	Wang et al. (2019)
	Chemical specific dermal permeability constant in water measured at 25 °C	*Kp*	cm/h	Chloroform: 0.16 BDCM: 0.18 DBCM: 0.2 Bromoform: 0.21	Wang et al. (2019)
	Exposure time considered during showering and bathing activities	*ET*	h	0.25	Legay et al. (2011)
Inhalation absorption	THM concentration in air	Ca	μg/L	Equation 8	Little (1992)
	Air intake rate	*Ira*	m^3^/h	0.83	Wang et al. (2019)
	Carcinogenic slope factor (Ingestion/dermal)	CSF	mg/kg/day	Chloroform: 0.0061 BDCM: 0.062 DBCM: 0.084 Bromoform: 0.0079	Lee et al. (2013)
	Carcinogenic slope factor (inhalation)	CSF	mg/kg/day	Chloroform: 0. 081 BDCM: 0.13 DBCM: 0.094 Bromoform: 0.0039	Lee et al. (2013)
Hazard index	Reference dose	RfD	mg/kg/day	Chloroform 0.01 (BDCM), 0.02 (DBCM), 0.02 (bromoform) 0.02	Abbas et al. (2015)

**Table 2:** Parameter values used in the modeling

Parameter	THMs	value	Reference
KOLA (L/min)	Chloroform BDCM DBCM Bromoform	7.4 5.9 4.6 3.7	Genisoglu et al. (2019)
QL (L/min)		5	Little (1992)
Vs (m^3^)		6	Genisoglu et al. (2019)
Qg (L/min)		50	Little (1992)
H at 40 °C	Chloroform BDCM DBCM Bromoform	0.350 0.186 0.102 0.058	
Kp (cm/h)	Chloroform BDCM DBCM Bromoform	0.16 0.18 0.20 0.21	Legay et al. (2011)
IRa (m^3^/h)	inhalation	20	

### Lifetime cancer risk through dermal absorption

THMs can enter to the body through showering, bathing, swimming, and other activities such as washing and handling water, but showering and bathing are most important (Arman et al. 2016). Average skin surface areas for males and females are 1.7 and 1.53 m^2^, respectively. Figures 1, 2 and 3 show the cancer risks of THMs through the dermal route of exposure for both males and females. Cancer risks of THMs through dermal contact for males and females in two sampling sites were 1 × 10^−6^ ≤ CR < 5.1 × 10^−5^, in the range acceptable low risk. Despite higher skin surface area in the males, the female has the highest cancer risk via the dermal route in the dormitory samples in contrast with other studies (Lee et al. 2004). Like oral ingestion, DBCM has the highest cancer risk followed by BDCM > bromoform > chloroform. Total lifetime cancer risks for the four THMs via dermal absorption for males were 5.32E-06 and 5.24E-06, and females were 6.45E-06 and 4.86E-06, respectively, in dormitory and school. In comparison with the two other routes, cancer risk through dermal absorption was not remarkable.

**Table 3:** Summary of THMs level (µg/L) before and after treatment by RO and ion exchange

Locality	*n*	Chloroform	BDCM	DBCM	Bromoform	Total THMs	Removal%
School, before T	5	5.6	18.26	37.6	36.64	98.1	
Dormitory, before T	5	21.2	25.2	34.4	21	101.8	
school, after T	5	0.84	2.2	3.4	2.44	8.88	90.9%
Dormitory, after T	5	1.82	3.9	6.2	3.04	14.96	85.3%

**Fig. 4:**
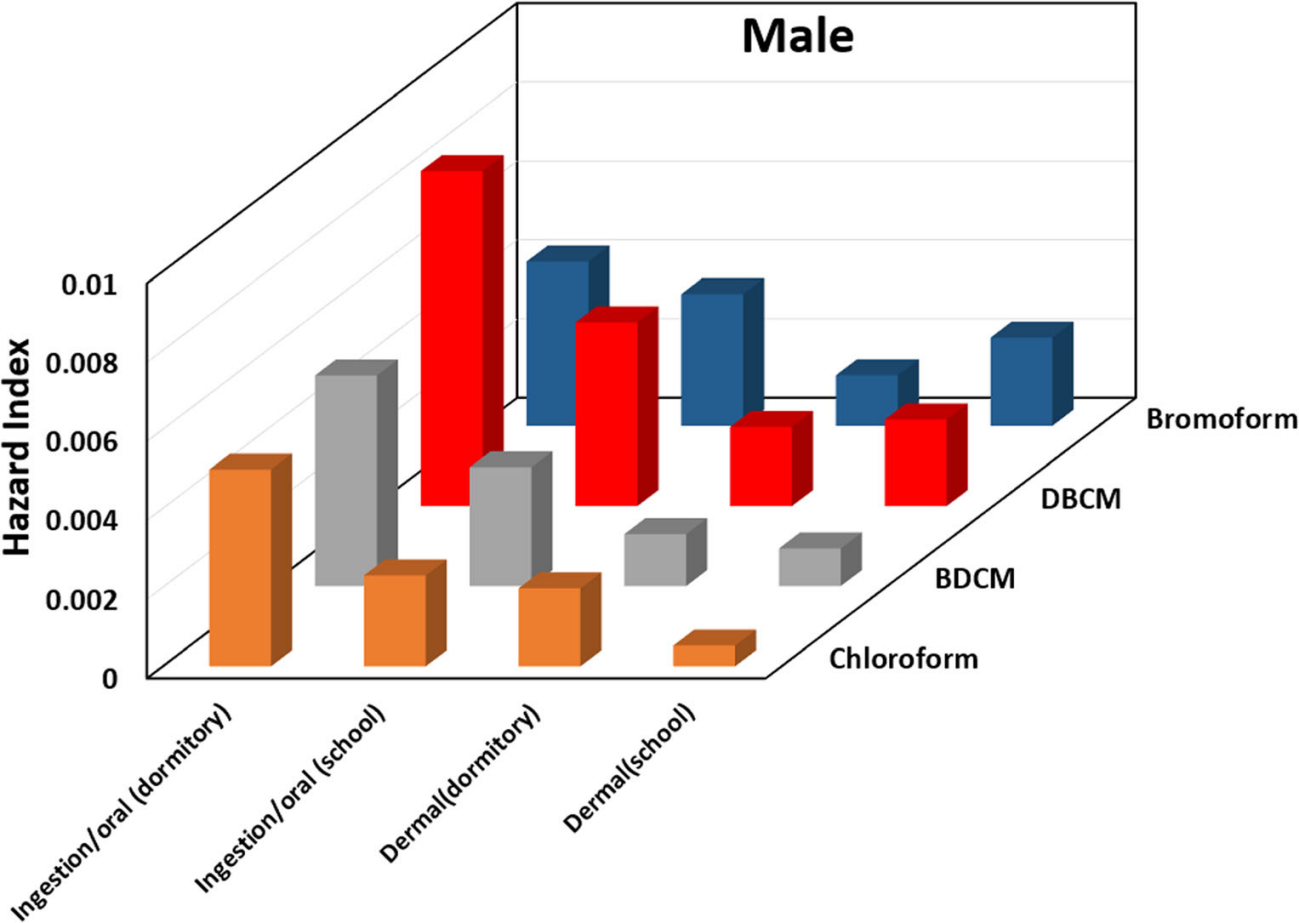
Hazard index of THMs through ingestion and dermal contact for males in dormitory and school

**Table 4:** General water quality parameters for different samples

Parameter	School, before T	School, after T	Removal%	Dormitory, before T	Dormitory, after T	Removal%
pH	7.22	7.16		7.04	7	
TOC	1.3	0.142	89%	0.82	0.108	86.8%
Br	0.38	0.03		0.38	0.058	
Cl_2_	0.3	0		0.76	0	

### Cancer risk from inhalation route

Eighty to ninety percent of the total risks due to exposure of THMs were dedicated to the inhalation exposure mainly through CHCl_3_ because this compound is highly volatile with low boiling point (Babaei et al. 2015; Siddique et al. 2015). The results of cancer risk through inhalation are shown in Figs. 1, 2and 3 in dormitory and school, respectively. Lifetime cancer risk for inhalation exposure for males were 1.78E-04 and 6.40E-05 and for females were 1.83E-04 and 6.59E-05 in dormitory and school, respectively. Lifetime cancer risk of total THMs through inhalation route for the four THMs was higher than USEPA unacceptable risk (1.0E_04) in the dormitory that means approximately 1 of every 10,000 individuals in Abadan could get cancer from the daily intake of water in their life. This value was in the acceptable high risk in school The major contributor through inhalation was CHCl_3_ with values of 87% followed by BDCM > DBCM > bromoform for dormitory and, in the school station, higher risk was related to CHCl_3_ with the value of 64%, because CHCl_3_ is highly volatile with the lower boiling point than other THMs, followed by DBCM > BDCM > bromoform. Some studies also reported similar results that the main route of exposure to THMs was inhalation (Babaei et al. 2015; Lee et al. 2013) (Table 5).

**Fig. 5:**
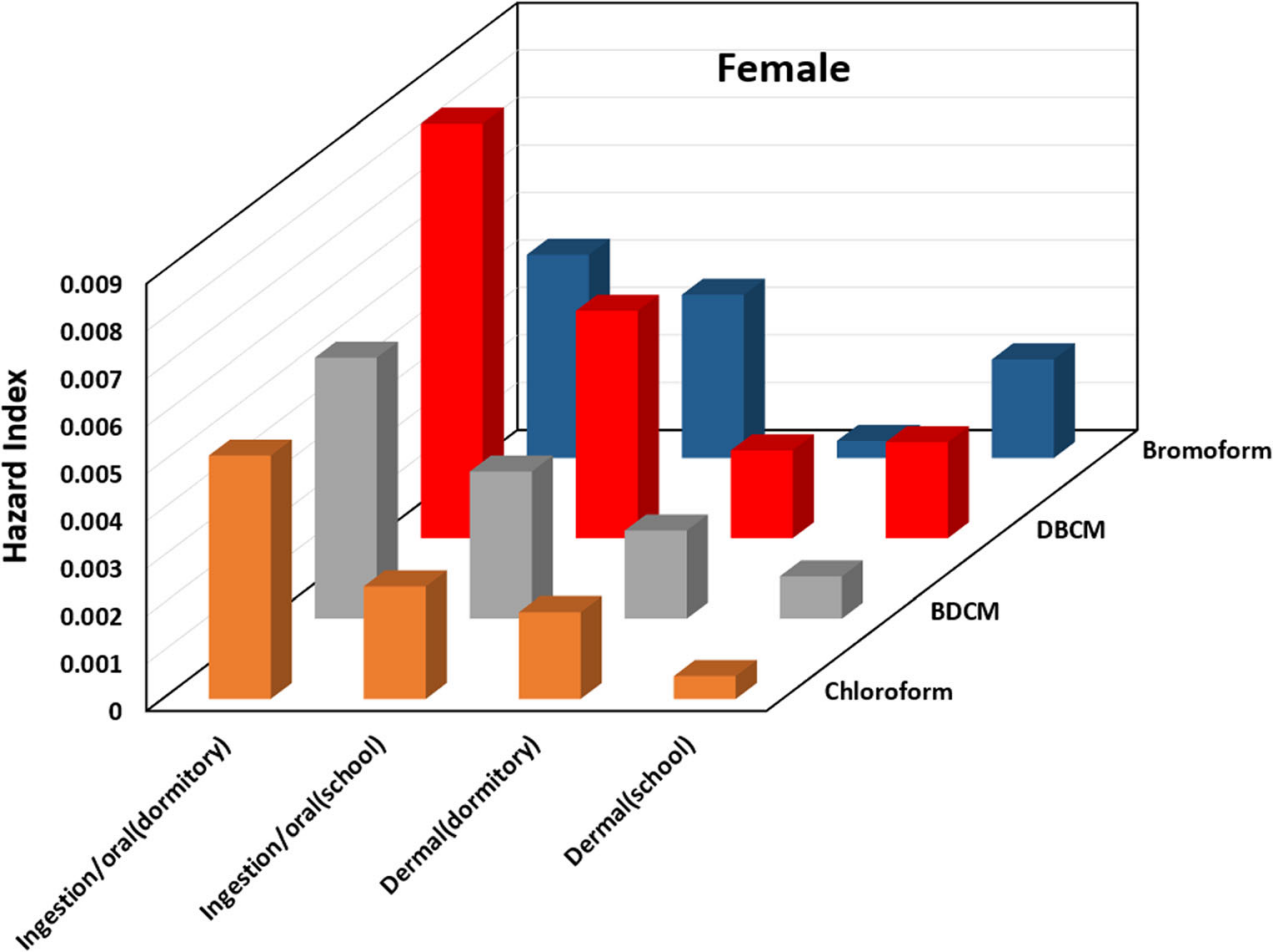
Hazard index of THMs through ingestion and dermal contact for females in dormitory and school

**Table 5:** Mean health risk of THMs through three exposure routes (ingestion, dermal contact, and inhalation) and the contribution of THM species

System	THMs	Oral	Dermal	Inhalation
Ion exchange (male)	Chloroform	3.03831E-07 2%	1.20331E-07 2%	0.000154246 87%
	BDCM	6.61741E-06 30%	1.63552E-06 31%	1.18211E-05 6%
	DBCM	1.42529E-05 65%	3.36092E-06 63%	8.36157E-06 5%
	Bromoform	6.57252E-07 3%	2.02608E-07 4%	3.19726E-06 2%
TTHMs		2.18E-05 11%	5.32E-06 2%	0.000178 87%
Cancer risk		Acceptable low risk	Acceptable low risk	Unacceptable risk
Ion exchange (female)	Chloroform	3.12869E-07 2%	2.92508E-08 1%	0.000158834 87%
	BDCM	6.81424E-06 30%	3.1148E-06 48%	1.21728E-05 6%
	DBCM	1.46768E-05 65%	3.1148E-06 48%	8.61029E-06 5%
	Bromoform	6.76802E-07 3%	1.87771E-07 3%	3.29236E-06 2%
TTHMs		2.25E-05 11%	6.45E-06 3%	0.000183 86%
Cancer risk		Acceptable low risk	Acceptable low risk	Unacceptable risk
RO (male)	Chloroform	1.4023E-07 1%	3.17854E-08 1%	4.07443E-05 64%
	BDCM	3.7329E-06 31%	1.1851E-06 22%	8.56563E-06 13%
	DBCM	7.81609E-06 64%	3.67356E-06 70%	9.13939E-06 14%
	Bromoform	5.27531E-07 4%	3.53502E-07 7%	5.57846E-06 9%
TTHMs cancer risk		1.22E-05 15%	5.24E-06 6%	6.40E-05 79%
Cancer risk		Acceptable low risk	Acceptable low risk	Acceptable high risk
RO (female)	Chloroform	1.44401E-07 1%	2.94578E-08 1%	4.19562E-05 64%
	BDCM	3.84393E-06 31%	1.09832E-06 22%	8.82042E-06 13%
	DBCM	8.04858E-06 64%	3.40455E-06 70%	9.41124E-06 14%
	Bromoform	5.43223E-07 4%	3.27615E-07 7%	5.74438E-06 9%
TTHMs cancer risk		1.26E-05 15% acceptable low risk	4.86E-06 6% acceptable low risk	6.59E-05 79% acceptable high risk

### Non-cancer risk assessment

Hazard index (HI) values for four THM compounds through ingestion and dermal contact for males and females at two stations are shown in Figs. 4 and 5 respectively. Similar to other studies (Babaei et al. 2015; Kumari et al. 2015; Wang et al. 2019), the results showed that the oral route has higher hazard index values than dermal way. The total hazard indexes for the oral for males and females were 2.29 × 10^−2^ and 2.36 × 10^−2^ in dormitory and 1.33 × 10^−2^ and 1.36 × 10^−2^ in school. Other studies revealed that the chloroform was the main contributor to non-cancer risk (Kumari et al. 2015; Lee et al. 2004) but DBCM has the highest average hazard index value for both males and females in this study. This was due to the fact that DBCM concentrations were the highest among other THMs. Dormitory drinking water samples has the greater total hazard index of 2.29 × 10^−2^ and 2.36 × 10^−2^ through oral ingestion for males and females. This is rational because THMs concentration in the raw and treated water of dormitory water was high. The total hazard index of THMs through oral ingestion for males was lower than females. The total average hazard index for oral ingestion and dermal contact was lower than acceptable toxicity (Pentamwa et al. 2013). Therefore, non-cancer risk due to THMs exposure can lead to neurobehavioral effects, jaundice, and enlarged livers (Amjad et al. 2013).

## Conclusions

This study was conducted to monitor the occurrence of THMs and their cancer risk assessment through ingestion, dermal contact, and inhalation exposure routes in two stations for males and females. The results showed that THMs concentration before treatment with household treatment devices was higher than the USEPA limit. CHBr_2_Cl had the highest concentration, and CHCl_3_ had the lowest concentration. The lifetime cancer risks caused by all routes were higher than 10^−6^, which is the negligible risk level defined by the USEPA. The lifetime cancer risk for total THMs was highest in the dormitory station for inhalation route. Higher cancer risk through dermal exposure was related to DBCM. Inhalation was the main route of exposure followed by ingestion and dermal contact. However, regarding to the Abadan location and providing better water quality, DBP precursors should be removed by coagulation-flocculation, granular activated carbon, membranes. It also suggested that other disinfectants, i.e., ozone and ultraviolet can be as alternative for chlorine. To control side-effects from exposure to THMs, an optimal dose of chlorine can be used, and complete monitoring of chlorinated disinfection by-products is required at multiple locations.
